# Interactions of natural health products with biomedical cancer treatments

**Published:** 2008-08

**Authors:** D. Seely, D. Oneschuk

**Keywords:** Complementary medicine, cam, natural health products, nhps, chemotherapy, radiotherapy, antioxidants, drug–herb interactions, pharmacology

## Abstract

The use of complementary and alternative medicine (cam), including the ingestion of natural health products (nhps), is common among cancer patients. Of concern to clinicians and patients alike is the possibility that cam, used concurrently with biomedical therapy, may interact poorly with that therapy, especially chemotherapy and radiotherapy. Proponents of nhps argue that taking such products can help to reduce the side effects of conventional therapy and can provide an additional anticancer effect. However, opponents insist that the potential for harm is too great to warrant the risk of concurrent administration. There are promising examples of specific nhps that may provide patient benefit even when given in close proximity both to chemotherapy and to radiotherapy, but unfortunately, in part because of a rather limited evidence base, caution is warranted when considering the issue of therapeutic interactions. Strategic application of nhps before or after conventional therapy may be considered; however, concurrent application should be avoided as a general principle until further evidence is available regarding specific interactions.

